# Thyroid cancer incidence in cohorts exposed in childhood to ^131^I released during the Windscale nuclear reactor accident at Sellafield, England, in 1957

**DOI:** 10.1007/s00411-024-01087-y

**Published:** 2024-08-16

**Authors:** Richard J. Q. McNally, Richard Wakeford, Kathryn J. Bunch, Louise Hayes, Sally Vernon, Polly-Anne Jeffrey, Lizz Paley, Alex Elliott

**Affiliations:** 1 Population Health Sciences Institute & Newcastle University Centre for Cancer, Sir James Spence Institute, Newcastle University, Royal Victoria Infirmary, Queen Victoria Road, Newcastle upon Tyne, NE1 4LP UK; 2 Centre for Occupational and Environmental Health, Faculty of Biology, Medicine and Health, The University of Manchester, Oxford Road, Manchester, M13 9PL UK; 3 Formally of the Childhood Cancer Research Group, Department of Paediatrics, University of Oxford, Headington, Oxford, OX3 9DU UK; 4 National Disease Registration Service, National Health Service England, 10 South Colonnade, Canary Wharf, London, E14 4PU UK; 5 School of Medicine, Dentistry and Nursing, University of Glasgow, Glasgow, G12 8QQ UK

**Keywords:** Ionising radiation, ^131^I, Thyroid cancer, Nuclear reactor, Accident, Risk

## Abstract

A fire in one of the Windscale nuclear reactors at Sellafield (Cumbria, England) in October 1957 released 1,800 TBq of ^131^I (half-life, 8 days) to atmosphere. Measurements of ^131^I activity in thyroids of exposed children showed typical thyroid doses of tens of milligray, but with some exceeding 100 mGy. Radiation exposure in childhood is known to increase the risk of thyroid cancer. Consequently, an investigation was conducted into whether raised numbers of thyroid cancer cases occurred in those exposed to ^131^I as young children in Cumbria. A database of Cumbrian births from 1950 onwards allowed cohorts of 56,086 births during 1950–1958 and 137,444 births during 1959–1980 to be constructed, periods including children potentially exposed and unexposed, respectively, to ^131^I. Three areas of Cumbria with different ^131^I contamination levels were identified from monitoring data, and births assigned to these three areas for the two periods of birth. Members of these six sub-cohorts were linked to incident thyroid cancer cases in Great Britain during 1981–2020 using national cancer registration databases, providing thyroid cancer incidence rates. Incidence rate ratios (IRRs), with the lowest contamination area as a reference, were computed. No IRR differed discernibly from unity. For births during 1950–1958, the IRR for the combined highest and intermediate ^131^I contamination areas was 0.68 (95% confidence interval: 0.24, 1.56), and no case of thyroid cancer was found in the small cohort born in the highest contamination area. In conclusion, no increased risk of thyroid cancer in those exposed to ^131^I as young children in Cumbria in 1957 was detected. This study adds to the evidence on the long-term risk of thyroid cancer following childhood exposure to low and moderate levels of ^131^I, such as occurred following the Fukushima nuclear accident in 2011.

**Supplementary Information:**

The online version contains supplementary material available at 10.1007/s00411-024-01087-y.

## Introduction

In the week commencing 7 October 1957, a fire developed in the core of a nuclear reactor (Windscale No. 1 Pile) at Sellafield, sited on the west coast of the current county of Cumbria in north-west England (Arnold 2007; Penney et al. 2017; Anon 1957; Dunster et al. 2007; Wakeford 2007, 2017; Jones 2008, 2016). The fire resulted in an uncontrolled atmospheric release of radioactive material from a 120 m tall chimney during 10–11 October (Garland and Wakeford 2007). The radioactive plume was initially carried by a light wind to the north-east until a change in direction of a strengthening wind sent most of the released radionuclides towards the south-east (Dunster et al. 2007; Chamberlain and Dunster 1958; Garland and Wakeford 2007; Nelson et al. 2006; Johnson et al. 2007). Of the radionuclides discharged, ^131^I was of greatest concern because it concentrates in the thyroid gland and radiation was known to cause thyroid cancer when exposure at high levels occurred at a young age (Simpson et al. 1955). Iodine-131 has a physical half-life of eight days so exposure to the 1,800 TBq of ^131^I released during the Windscale accident (Garland and Wakeford 2007) only occurred within a few months of its discharge.

Immediately after the discharge, environmental monitoring established that ^131^I concentrations in milk were sufficiently high to require a ban on the distribution of locally-sourced milk in the most contaminated area of Cumbria to the south-east of Sellafield (Dunster et al. 2007; Loutit et al. 1960; Crick and Linsley 1984; Chamberlain and Dunster 1958). The limit set for the ban was a measured ^131^I activity concentration in milk of 3.7 kBq.L^− 1^ (0.1 mCi.L^− 1^), which was selected to restrict the absorbed dose to the thyroid of a child to less than 200 mGy (Dunster et al. 2007). In the worst affected area, the Cumbrian coastal strip extending around 20 km south-east of Sellafield, the milk distribution ban was in place for approximately six weeks (Dunster et al. 2007). In all, some three million litres of milk were disposed of via drains (Jackson and Jones 1991).

Monitoring for ^131^I activity in the thyroids of 125 members of the public, of whom 29 were children, resident in this downwind sector found that typical thyroid doses were several milligray for adults and a few tens of milligray for children (Dunster et al. 2007; Loutit et al. 1960; Crick and Linsley 1984). However, in some instances thyroid doses in children approached 100 mGy and beyond (the maximum recorded was 160 mGy), indicating that efforts to control the ingestion of contaminated milk had not been fully effective (Loutit et al. 1960; Crick and Linsley 1984). In the absence of the consumption of locally-sourced milk, inhalation of ^131^I would have been the main contributor to the measured thyroid activities (Crick and Linsley 1984; COMARE 2016). Thyroid monitoring of 113 people living outside the most contaminated area found either a zero thyroid dose (107) or a very low dose (the maximum was 12 mGy) (Loutit et al. 1960). Although the thyroid monitoring programme sought to measure ^131^I activity in the thyroids of those individuals who may have consumed contaminated milk, as well as of those considered to be more representative of the downwind population (Loutit et al. 1960), there are likely to have been others who had drunk locally-sourced milk whose thyroid doses had not been measured, and it has been estimated that such doses could have been up to 360 mGy (Crick and Linsley 1984).

Epidemiological studies have demonstrated that following radiation exposure of the thyroid, the risk of thyroid cancer is notably greater when exposure occurs in childhood; the excess risk persists for many years into adult life (NCRP 2009; UNSCEAR 2013; EPA 2011; COMARE 2016; Furukawa et al. 2013; Veiga et al. 2016; Lubin et al. 2017; Boice 2006). Further, the radiation-related risk has been modelled to be manifest as a proportional increase in the baseline risk (Berrington de Gonzalez et al. 2012), although uncertainties in this assumption are inevitable (UNSCEAR 2020, EPA 2011). Consequently, an increase in the risk of thyroid cancer incidence resulting from an intake by a child of ^131^I from the 1957 Windscale accident is likely to persist for decades and follow the attained age distribution of the baseline incidence of thyroid cancer.

Crick and Linsley (1984) estimated that the collective thyroid dose received in Europe from ^131^I released by the Windscale fire was 2.6 × 10^4^ person Gy. Clarke (1989, 1990) tentatively suggested that perhaps 60 or so cases of thyroid cancer might eventually result from this collective thyroid dose. Of this collective thyroid dose, ~ 95% was received by the population of the UK and 3.2 × 10^3^ person Gy in Cumbria within 50 km of Sellafield (Crick and Linsley 1984). This estimate for Cumbria makes the assumption that the local milk distribution ban was completely effective, which reduces the collective thyroid dose for Cumbria to ~ 50% of what would have been received otherwise (Crick and Linsley 1984). The implication (Clarke 1990) is that around eight cases of thyroid cancer would occur among those residing in affected areas of Cumbria at the time of the accident; most would be expected to occur among those exposed as young children (Ron et al. 2012). Repeating these calculations using a later risk coefficient (ICRP 2007) implies a one-third increase in these predicted numbers of cases.

Epidemiological studies of cancer in Cumbria have not found excesses of thyroid cancer incidence that can be attributed to ^131^I released during the Windscale fire (Bunch et al. 2014; COMARE 2016; McNally et al. 2016, 2017). However, these studies have not focused upon the incidence of thyroid cancer in those most at risk from intakes of ^131^I from the accident: children living in the most contaminated areas of Cumbria during mid-October 1957 who would have received the highest thyroid doses, particularly if they had consumed locally-sourced milk. In this study thyroid cancer incidence was examined among the cohort born during 1950–1958 in those areas of Cumbria most affected by ^131^I contamination from the Windscale accident. This study was conducted following a recommendation by the UK Committee on Medical Aspects of Radiation in the Environment (COMARE) (COMARE 2016).

## Materials and methods

### Cohort construction

#### Calendar period of birth

During the early-1990s, an electronic database of Cumbrian births was created at Newcastle University for the purposes of epidemiological research (Parker et al. 1997). This database included, for each birth from 1950 onwards to mothers usually resident in Cumbria, the name, sex, date of birth and maternal residential address, and was constructed from publicly available birth records; a postcode and the grid reference of the postcode centroid were assigned to the maternal address at the birth of the child (Parker et al. 1997).

Using the Cumbrian births database (Parker et al. 1997), two cohorts of births during 1950–1958 and 1959–1980 were produced. Births during the first period included those who were young children, or *in utero*, in mid-October 1957 and had the potential of being exposed to ^131^I released during the Windscale accident. Those born during the second period would have been unexposed to ^131^I discharged during the reactor fire because all the ^131^I emitted would have undergone radioactive decay to stable ^131^Xe (^131^I *t*_½_ = 8 days). The cohort of Cumbrian births during 1959–1980 was used to examine whether background thyroid cancer risk factors may have differentially influenced incidence rates in those areas most affected by radioactive contamination compared to those areas least affected, and also whether there was any effect of discharged radionuclides (for example, ^210^Po and ^137^Cs) that persisted in the environment for longer than several months, although thyroid doses will have been dominated by intakes of ^131^I (Crick and Linsley 1984).

#### Geographical area of birth

**Fig. 1:**
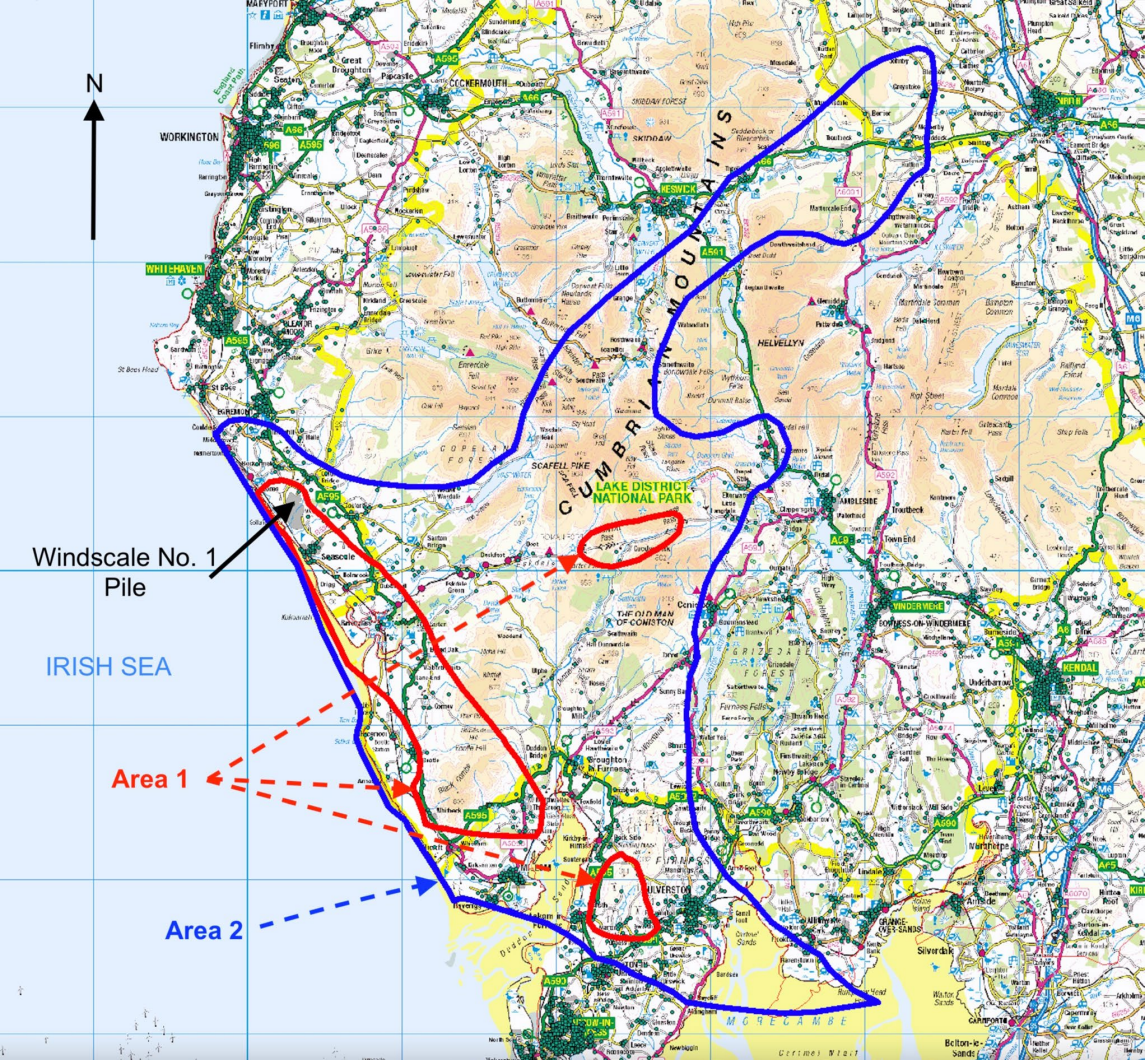
Map of the south-western part of the present-day county of Cumbria (north-west England) showing the three areas with different levels of ^131^I contamination from the 1957 Windscale reactor fire used in the study: Area 1, with the highest contamination, is enclosed by a red contour (three separate areas), and Area 2, with the intermediate level of ^131^I contamination, is enclosed by a blue contour. The remainder of Cumbria, Area 3, had the lowest level of ^131^I contamination. Contours are based on maps of ^131^I contamination levels published in the late-1950s (Chamberlain 1959; Booker 1958). Green dots show postcode centroids, used to allocate births to a particular contamination area. Map provided via OS Digimap (https://digimap.edina.ac.uk/roam/map/os) with data overlay via ArcGIS Desktop (https://www.esri.com/en-us/arcgis/products/arcgis-desktop/overview)

Comprehensive environmental monitoring following the release of ^131^I during the Windscale accident provided information on the geographical distribution of ^131^I deposited on the ground, as measured in grass and soil, and in milk (Dunster et al. 2007; Chamberlain and Dunster 1958; Loutit et al. 1960), and particularly detailed maps of the results of ^131^I monitoring in Cumbria were published in the late-1950s (Chamberlain 1959; Booker 1958). These maps permitted the construction, via the geographic information system (GIS) ArcGIS Desktop (https://www.esri.com/en-us/arcgis/products/arcgis-desktop/overview, accessed 1 May 2019), of two boundaries dividing Cumbria into three areas: Area 1, with the highest levels of ^131^I contamination; Area 2, with intermediate levels of ^131^I contamination; and Area 3, the remainder of Cumbria with the lowest levels of ^131^I contamination. These two boundaries broadly correspond to areal ^131^I activity concentrations of 185 kBq.m^− 2^ (5 µCi.m^− 2^) and 37 kBq.m^− 2^ (1 µCi.m^− 2^), respectively (Chamberlain 1959; Booker 1958), and ^131^I activity concentrations in locally produced milk of 18.5 kBq.L^− 1^ (0.5 µCi.L^− 1^) and 1.85 kBq.L^− 1^ (0.05 µCi.L^− 1^), respectively (Booker 1958). A measured ^131^I activity concentration in milk of 3.7 kBq.L^− 1^ (0.1 µCi.L^− 1^) was the limit above which the distribution of milk was prohibited (Dunster et al. 2007), but it was decided that Area 2 should extend beyond the area covered by the milk distribution ban because, as noted by Jackson and Jones (1991), later protection criteria would have led to milk supply restrictions in a larger area. Area 1 (with the highest ^131^I contamination) consists of a main area and two separate small areas because of differences in local ^131^I deposition (Chamberlain 1959; Booker 1958). Figure 1 presents a map of the location of these boundaries in Cumbria.

To assign each Cumbrian birth to a contamination area, the centroid of each postcode in Cumbria was plotted onto the output of the GIS contours using the grid references of these centroids. Births during 1950–1958 and 1959–1980 to mothers with a residential address in Cumbria, with an associated postcode and centroid grid reference, were then assigned to the three areas of different ^131^I contamination levels, resulting in six non-overlapping sub-cohorts of Cumbrian births. Figure 1 shows the geographical distribution of Cumbrian postcode centroids with respect to the contamination areas.

### Identification of thyroid cancer cases

Individual members of the six sub-cohorts were linked to the national cancer registration databases of England, Wales and Scotland to achieve coverage of incident cases of thyroid cancer throughout Great Britain. Deaths (and, where available, emigrations from Great Britain) among the sub-cohorts were also ascertained for the purposes of computing person-years of follow-up. The Welsh Cancer Intelligence and Surveillance Unit kindly gave permission for Welsh data to be included in the linkage exercise for England.

Initial linkage with the English cancer registry (Henson et al. 2019), which uses current National Health Service (NHS) numbers in the identification of individuals, revealed a major problem whereby those who had changed their name between birth and the 1995 introduction of current NHS numbers could not be identified with certainty because sufficient earlier data were not routinely available. This particularly affected women who married prior to 1995. However, for their study of the incidence of a range of types of cancer in Cumbria during various periods, Bunch et al. (2014) made use of, *inter alia*, the Cumbrian births database. Bunch et al. (2014) conducted a detailed exercise of providing additional personal information for members of the Cumbrian births database, using data held by the then Childhood Cancer Research Group at the University of Oxford, that would, whenever possible, enable unambiguous linkage to individuals in the English national database. One of the authors (K.J.B.) was able to make use of additional data from this enhanced Cumbrian births database in the current linkage exercise. Such linkage problems did not occur for the Scottish registry because sufficient historical data were available in the system.

Details of individual members of the six sub-cohorts were sent to the English National Disease Registration Service (NDRS) and the Scottish Cancer Registry (SCR) for linkage to registrations of thyroid cancer in England and Wales and in Scotland, respectively. Numbers of cases of thyroid cancer incident in the six sub-cohorts during the 40-year period 1981–2020 (divided into 5-year follow-up periods to avoid confidentiality problems associated with small numbers of cases) were supplied by the two organisations. Only numbers of cases in the six sub-cohorts and 5-year follow-up periods were provided; no other details that could potentially allow identification of the affected individuals were supplied. The first year for thyroid cancer registrations was 1981 because this was the first year that registrations were judged to be effectively complete throughout Great Britain by the national registries, and the last year was 2020 because this was the last year of complete registrations at the time of the matching exercises.

Incident cases of all malignant neoplasms of the thyroid gland (ICD-10 code C73, and equivalent earlier codings, e.g., ICD-9 code 193) were included in the study.

### Calculation of incidence rates and incidence rate ratios

Person-year (P-Y) data were provided by NDRS, which took account of deaths in the sub-cohorts in England and Wales. However, these P-Y data did not incorporate deaths in Scotland, so they were corrected using the numbers of deaths in each sub-cohort during each 5-year period of follow-up supplied by SCR. Follow-up in Scotland of those born in Cumbria who migrated to Scotland accounts for 2.5–5% of the total P-Ys of follow-up, depending on the sub-cohort.

Thyroid cancer incidence rates (cases per 10^5^ P-Y) were calculated for each of the six sub-cohorts together with incidence rate ratios (IRRs) for Areas 1 and 2, with the rate in Area 3 (the lowest level of ^131^I contamination) used as a reference rate. Mid-P exact 95% confidence intervals (Berry and Armitage 1995) for incidence rates and IRRs were computed using the OpenEpi statistics package (www.OpenEpi.com, accessed 20 November 2023).

Originally, the intention was to calculate sex-specific thyroid cancer incidence rates for each of the six birth sub-cohorts, but difficulties arose from the limited numbers of sex-specific cases, which posed problems of confidentiality. Consequently, thyroid cancer incidence rates were calculated for both sexes combined. Given the higher thyroid cancer incidence rates experienced by females, particularly in young middle age (CRUK 2023a), the proportions of female births in the sub-cohorts born in the highest and intermediate contamination areas were compared with those in the reference sub-cohorts born in the lowest contamination area, to ensure that biases had not been introduced through the use of rates for both sexes combined. This comparison exercise showed that the proportions of female births in contamination Areas 1 and 2 did not differ significantly from those in reference Area 3 (Supplementary Table S1), so the use of rates for combined sexes is considered to be acceptable.

## Results

Of 206,703 livebirths during 1950–1980 registered to a mother resident in Cumbria and recorded as a unique entry in the Cumbrian births database, 193,530 (93.6%) with a maternal residence at birth assigned to one of the three contamination areas could be linked unambiguously to an NHS number. This represents 56,086 births during 1950–1958 and 137,444 during 1959–1980 (respectively, 91% and 95% of livebirths with a known Cumbrian postcode). A breakdown of these linkages by birth cohort and contamination area is given in Supplementary Table S2.

The numbers of cases of thyroid cancer recorded as incident in Great Britain during 1981–2020 among those born during the 9-year period 1950–1958 (a total of 85 cases) and the 22-year period 1959–1980 (182 cases) to mothers resident in the three areas of Cumbria with different levels of ^131^I contamination in mid-October 1957 are shown in Table 1. Also presented in Table 1 are the total numbers of livebirths in each of the six sub-cohorts and the person-years of follow-up during 1981–2020, providing thyroid cancer incidence rates (cases per 10^5^ P-Y) for each of the sub-cohorts. Births in Area 3 make up 91.6% of Cumbrian births in both periods.

**Table 1:** Numbers of livebirths, person-years (P-Y) of follow-up and cases of thyroid cancer incident in Great Britain during 1981–2020, and the resulting thyroid cancer incidence rates and incidence rate ratios, for births in Cumbria during the two periods, 1950–1958 and 1959–1980, to mothers resident in three areas contaminated to different levels with ^131^I from the 1957 Windscale fire (Fig. 1). Follow-up is in England and Wales and in Scotland during 1981–2020. Confidence intervals are Mid-P exact. See main text and supplementary material for details

Area of ^131^I Contamination Level in Cumbria	Number of Livebirths	Person-Years (P-Y) of Follow-up	Number of Cases of Thyroid Cancer Incident in Great Britain, 1981-2020^a^	Incidence Rate of Thyroid Cancer, cases per 10^5^ P-Y (95% Confidence Interval)	Thyroid Cancer Incidence Rate Ratio (95% Confidence Interval)
Livebirths in Cumbria during 1950–1958 (exposed to various levels of ^131^I from the 1957 Windscale accident)					
Area 3 (lowest contamination; reference area)	51,377	1,995,350	80^b^	4.01 (3.20, 4.96)	1 (reference incidence rate)
Area 2 (intermediate contamination)	4,066	158,295	5	3.16 (1.16, 7.00)	0.79 (0.28, 1.81)
Area 1 (highest contamination)	643	25,260	0	0 (0, 11.86)	0 (0, 3.01)
Areas 1 and 2 combined	4,709	183,555	5	2.72 (1.00, 6.04)	0.68 (0.24, 1.56)
Livebirths in Cumbria during 1959–1980 (unexposed to ^131^I from the 1957 Windscale accident)					
Area 3 (reference area)	125,903	4,980,160	163^c^	3.27 (2.80, 3.81)	1 (reference incidence rate)
Area 2	10,171	402,820	18	4.47 (2.73, 6.93)	1.37 (0.82, 2.18)
Area 1	1,370	54,240	1^b^	1.84 (0.09, 9.09)	0.56 (0.03, 2.81)
Areas 1 and 2 combined	11,541	457,060	19	4.16 (2.58, 6.37)	1.27 (0.77, 2.00)

^a^ All cases were registered in England except for:

^b^ one case in each of the two relevant sub-cohorts was registered in Wales, and

^c^ four cases were registered in Scotland

IRRs for the highest contamination Area 1 and intermediate contamination Area 2, with the incidence rate for lowest contamination Area 3 used as the reference rate, are 0 (95% CI: 0, 3.01) and 0.79 (95% CI: 0.28, 1.81), respectively, for those born during 1950–1958 who had the potential of being exposed to ^131^I from the 1957 Windscale accident (Table 1). Indeed, no case of thyroid cancer was recorded during 1981–2020 among those born in the highest contamination Area 1, but the number of livebirths in this sub-cohort was only 643 with the consequence that the confidence interval for the IRR is wide. The IRR for Areas 1 and 2 combined is 0.68 (95% CI: 0.24, 1.56) (Table 1).

IRRs for Areas 1 and 2 for individuals born during 1959–1980 who were unexposed to ^131^I from the accident are also unremarkable (Table 1); the IRR for Areas 1 and 2 combined is 1.27 (95% CI: 0.77, 2.00). This provides little indication of differences in background thyroid cancer risk factors between these two areas and reference Area 3.

## Discussion

### Key finding

The ratios of the rates of thyroid cancer incidence during 1981–2020 among those born during 1950–1958 to mothers living in those areas of Cumbria that experienced the highest and intermediate levels of ^131^I contamination following the fire in one of the Windscale nuclear reactors at Sellafield in 1957 are 0 (95% CI: 0, 3.01) and 0.79 (95% CI: 0.28, 1.81), respectively, when compared with the equivalent rates for births in the remainder of Cumbria where contamination levels were lowest. These incidence rate ratios provide little evidence for ^131^I released during the accident influencing the risk of thyroid cancer among those who were young children in 1957.

### Strengths of the study

This cohort study has been able to identify the great majority (90.5%) of livebirths during 1950–1958 to mothers resident in Cumbria in sufficient detail to permit their follow-up for thyroid cancer incidence during the 40-year period 1981–2020 throughout Great Britain. A total of 56,086 births during 1950–1958 were assigned to three areas with different measured levels of ^131^I contamination from the Windscale nuclear reactor fire of October 1957. Those born in the highest (Area 1) and intermediate (Area 2) contamination areas included those exposed to ^131^I from the accident as young children (or *in utero*), which poses the greatest risk of consequent thyroid cancer (Ron et al. 2012). The limited numbers of births during 1950–1958 in these two areas (642 and 4,066, respectively) results in small numbers of incident thyroid cancer cases (0 and 5 observed, respectively) and imprecise incidence rates. Nonetheless, incidence rate ratios, with the lowest (Area 3) contamination area incidence rate (based on 51,377 births and 80 thyroid cancer cases) used as a reference, are less than unity in both these contaminated areas (Table 1). The upper 95% confidence limit of the rate ratio for the combined Areas 1 and 2 (Table 1) implies that it is unlikely that the risk of thyroid cancer incidence among those born during 1950–1958 in the ^131^I-contaminated areas is greater than 1½ times that for those born in the remainder of Cumbria.

Of Cumbrian livebirths during 1959–1980, 95% (137,444) could be followed-up for thyroid cancer incidence during 1981–2020. Members of this birth cohort were not exposed to ^131^I from the Windscale fire because the ^131^I had radioactively decayed. IRRs for Areas 1 and 2 are still based on small numbers of cases (1 and 18 observed, respectively), but there is little indication that thyroid cancer incidence rates in these two areas differ from the reference rate for Area 3; had such differences been found this might have suggested that background factors needed to be considered in assessing findings for births during 1950–1958.

### Limitations of the study

Excluded from the follow-up are those individuals in the Cumbrian births database with insufficient personal details to permit unambiguous identification in the national databases. For births during 1950–1958, such excluded individuals account for 9.5% of entries in the Cumbrian births database, and less than this for those known to have been born in Area 1 (7%) and Area 2 (8%). For births during 1959–1980, the overall percentage of untraceable individuals is 5%. Therefore, those persons omitted from follow-up represent relatively small proportions of the birth cohorts, and it is unlikely that their exclusion would substantially affect results.

Those born in Cumbria before 1950 could not be identified because the Cumbrian births database only started with births in 1950 (Parker et al. 1997). However, those included in the study would have been 0–7 years of age (or *in utero*) at the time of the 1957 accident and it is known that young children are at the highest risk of future thyroid cancer following radiation exposure (UNSCEAR 2013; NCRP 2009; COMARE 2016; EPA 2011; Boice 2006), so persons born during 1950–1958 include those most at risk of thyroid cancer after intake of ^131^I released during the fire. By way of illustration, using the radiation risk model for thyroid cancer incidence developed by the US National Cancer Institute (Berrington de Gonzalez et al. 2012), for a typical person receiving a constant thyroid dose rate throughout their life, about 50% of the lifetime thyroid cancer risk is attributable to doses received at ages 0–7 years. However, this particular model may underestimate the contribution from the exposure of young children to the overall risk (EPA 2011), a conclusion supported by recent studies (Furukawa et al. 2013; Veiga et al. 2016; Lubin et al. 2017). Further, the thyroid dose per unit intake of ^131^I activity is notably greater for children, particularly young children (ICRP 1989; Singh et al. 2023), leading to higher expected thyroid doses in young children, as observed in the measurements of thyroid ^131^I activities in 1957 (Loutit et al. 1960). Consequently, follow-up for thyroid cancer incidence among those born during 1950–1958 would be expected to detect most of the Cumbrian-born cases related to the Windscale fire.

Follow-up of the sub-cohorts only commenced in 1981, when thyroid cancer case ascertainment was considered by the national cancer registries to be effectively complete throughout Great Britain. Therefore, cases of thyroid cancer could have been incident in the ^131^I-exposed sub-cohorts before 1981. However, the minimum latent period for thyroid cancer is approximately 5 years (with a likely range of 3–7 years) (Berrington de Gonzalez et al. 2012; UNSCEAR 2020) so even with a minimum latent period of 3 years, cases related to radiation exposure in late-1957 would not occur until late-1960 at the earliest. Supplementary Table S4 of McNally et al. (2016) presented, for attained age groups of 0–9 years and 10–24 years, the numbers of incident cases of, and deaths from, thyroid cancer in four sub-periods during 1961–1980 and 1959–1980, respectively, recorded as resident at diagnosis/death in two areas of west and south Cumbria that largely include contamination Areas 1 and 2. Only one incident case was recorded, diagnosed during 1961–1965 in the 0–9 year age group (Cook-Mozaffari et al. 1987), and that child lived in the area that includes part of the neighbouring county of Lancashire as well as south Cumbria (and the child was almost certainly not resident in Area 1 at diagnosis) (McNally et al. 2016). Further, this child could have been born after 1958, with no potential for exposure to ^131^I from the accident. Additionally, Bunch et al. (2014) identified just two cases of thyroid cancer incident in Great Britain during 1971–1980 among births from 1950 onwards in the whole of Cumbria, and one of the authors (K.J.B.) can report that both these cases occurred among births outside west Cumbria, so it is very unlikely that these two individuals were born to mothers resident in Area 1. It is possible that cases could have been incident in the 1960s among the sub-cohorts born during 1950–1958 in Areas 1 or 2, who had emigrated from these ^131^I-contaminated areas of Cumbria prior to diagnosis, but anything other than a small number of such cases seems implausible. Overall, from the very small numbers of cases incident before 1981 that are presented here, it is most unlikely that cases diagnosed prior to 1981 would have a material impact upon the findings of this study.

Follow-up covered 40 years and ended in 2020, when members of the sub-cohorts born during 1950–1958 would have been 62–70 years of age. Therefore, cases of thyroid cancer could be incident in the ^131^I-exposed sub-cohorts after 2020. However, currently, those who have attained an age of 62–70 years in the UK will have experienced around 80% of their baseline lifetime risk of thyroid cancer incidence (CRUK 2023a), so substantial numbers of cases diagnosed after 2020 would not be expected. Further, there is evidence that following radiation exposure in childhood the relative risk of thyroid cancer decreases with attained age, although still remaining raised (Veiga et al. 2016; Lubin et al. 2017; EPA 2011; NCRP 2009; Furukawa et al. 2013; Boice 2006), which would reduce the expected risk of radiation-related thyroid cancer incidence beyond 2020.

This study cannot identify those born in Cumbria during 1950–1958 who had moved from their maternal residence at birth before the second week of October 1957. Migration between different contamination areas will lead to assignment to an area that does not correspond to residence at the time of the accident, and emigration from Cumbria of those born in Areas 1 and 2 will lead to their incorrect inclusion in the follow-up as exposed to ^131^I. Similarly, those born in the last quarter of 1958 to a mother living in the contaminated areas would have been conceived after the discharged ^131^I had decayed so would have been unexposed, and such individuals are also included in the follow-up as exposed; in addition, the thyroid doses received during the pre-fetal stages of intrauterine development from maternal intakes of ^131^I are very small (ICRP 2001) so those born during the third quarter of 1958 are effectively unexposed. Further, the study cannot identify those living in Cumbria during mid-October 1957, but born during 1950–1957 outside the county. Therefore, in this birth cohort study there is an inevitable risk of misclassification of ^131^I exposure, and also the omission of individuals born outside Cumbria who resided in contaminated areas at the time of the accident. However, the inclusion of births during 1950–1958 to a mother resident in the contaminated areas would be expected to capture the great majority of those exposed to ^131^I from the accident and to include relatively few unexposed individuals.

The ^131^I contamination contours selected for this study were judged to be the best that could be derived from the available evidence, but they are geographical boundaries that cannot perfectly reflect ^131^I exposure of individuals, particularly exposure related to personal habits, such as drinking locally-sourced milk. Consequently, these geographical divisions must produce some exposure misclassification. Even so, data on spatial ^131^I contamination are quite detailed (Chamberlain 1959; Booker 1958), and the thyroid monitoring programme demonstrated that the highest thyroid doses were found in the predicted neighbourhoods (Loutit et al. 1960).

The incidence rates of thyroid cancer during 1981–2020 among those born in reference Area 3 during 1950–1958 and 1959–1980 are 4.01 (95% CI: 3.20, 4.96) per 10^5^ P-Y and 3.27 (95% CI: 2.80, 3.81) per 10^5^ P-Y, respectively (Table 1). These incidence rates are broadly compatible with what would be expected from (sex-averaged) national rates for births in the 1950s and 1960s/1970s (COMARE 2016; CRUK 2023b, c), although McNally et al. (2016) found that thyroid cancer incidence rates for Cumbria as a whole tended to be somewhat higher than those for the rest of England for births during these periods. The temporal pattern of these Cumbrian incidence rates, however, did not suggest an influence of the 1957 Windscale fire (McNally et al. 2016, 2017).

A complication in predicting Cumbrian thyroid cancer incidence rates from national data is the increase in incidence that has been apparent over the 40-year period 1981–2020 in the UK (McNally et al. 2012; Oke et al. 2018; dos Santos Silva and Swerdlow 1993); Cancer Research UK has estimated that the European age-standardised incidence rate increased by 175% in the UK between 1993–1995 and 2016–2018, which was of a similar size for males and females (CRUK 2023b, c). This increase in incidence may not have been geographically or temporally uniform in the UK, as discussed by McNally et al. (2016, 2017). Rates of incidence of thyroid cancer have increased in many countries (Furuya-Kanamori et al. 2016; Huang et al. 2023; Kim et al. 2020; Schuster-Bruce et al. 2022; La Vecchia et al. 2015), and much of this has been attributed to “overdiagnosis” (the diagnosis of a medical condition that would never have caused any symptoms or problems (Brodersen et al. 2018)) (Vaccarella et al. 2016; Li et al. 2020; Ahn et al. 2014; Wakeford et al. 2016; Zaridze et al. 2021; Furuya-Kanamori et al. 2016), although some of the reported increases may not be entirely attributable to overdiagnosis (van Gerwen et al. 2022; Kitahara and Sosa 2016). Overdiagnosis of thyroid cancer has been strikingly demonstrated by Furuya-Kanamori et al. (2016), who conducted a meta-analysis of 35 studies of a total of 12,834 autopsies with no known history of thyroid pathology. They found a “substantial reservoir” of incidental differentiated thyroid cancer that had not risen over several decades and concluded that the reported increasing incidence of thyroid cancer “is related to increasing detection of stable incidental disease” (Furuya-Kanamori et al. 2016). Concern about overdiagnosis of thyroid cancer has led to a recommendation that unselected population monitoring of thyroid health should not take place after a nuclear accident (Togawa et al. 2018), although there is no evidence of any screening effect in Cumbria after the Windscale accident.

### Other sources of radiation exposure in Cumbria

Although in terms of the risk of thyroid cancer ^131^I was the most important radionuclide to be released during the 1957 Windscale fire, other radionuclides discharged, e.g., ^210^Po (*t*_½_ = 138 days) and ^137^Cs (*t*_½_ = 30 years), would have delivered smaller doses to the thyroid (Crick and Linsley 1984). These radionuclides with longer half-lives than ^131^I (*t*_½_ = 8 days) would have led to exposure over more protracted periods. However, the geographical distribution of contamination by these other radionuclides was much the same as that of ^131^I (Booker 1958), so that Areas 1 and 2 also provide an indication of contamination levels from these radionuclides. For those born during 1959–1980, who would have been unexposed to ^131^I but to some extent exposed to longer-lived radionuclides, there is little evidence that births to mothers living in Areas 1 and 2 have an increased incidence of thyroid cancer relative to births in Area 3 (Table 1).

Those born in west Cumbria during 1950–1958 would have been exposed to ^131^I from sources other than the Windscale fire, including other discharges from Sellafield and atmospheric nuclear weapons testing (COMARE 2016). For a young child resident during October 1957 in Seascale, a village situated 3 km south of Sellafield, the dose to the thyroid from the Windscale accident has been assessed to be about 25 mGy, which assumes that locally-sourced milk was not consumed (COMARE 2016). This is comparable to the thyroid dose accumulated over 25 years by children in Seascale born in the 1950s from routine discharges of ^131^I from Sellafield, who also received over this period a total thyroid dose of around 2 mGy from atmospheric nuclear weapons testing fallout (COMARE 2016). By comparison, the cumulative equivalent dose to the thyroid from natural background radiation was 26 mSv (COMARE 2016). An additional dose to the thyroid from ^131^I was received from fallout from the Chornobyl reactor accident in Ukraine in mid-1986, when doses to young children in Cumbria would have been a few milligray (COMARE 2016). Further details of thyroid doses in Cumbria can be found elsewhere (COMARE 2016; McNally et al. 2016).

### Studies of exposure to ^131^I

There is a clear excess incidence of thyroid cancer among those highly exposed as young children to ^131^I discharged during the severe nuclear reactor accident at Chornobyl in April 1986, when 1,800 PBq of ^131^I was released. Tens of thousands of children in the worst affected areas of the former USSR received thyroid doses in excess of 1 Gy, mainly as a consequence of drinking heavily contaminated milk (UNSCEAR 2011, 2018). Away from the highly contaminated areas an excess risk of thyroid cancer is far from clear (UNSCEAR 2011; Auvinen et al. 2014; Zaridze et al. 2021).

An historical cohort study of thyroid disease in relation to 27 PBq of ^131^I released to atmosphere from the Hanford Nuclear Site in Washington State during 1944–1957 included 3,440 people potentially exposed as children. The estimated mean thyroid dose for the cohort was 174 mGy. Little evidence was found for a radiation-related increased risk of thyroid cancer incidence (based on 19 cases) (Davis et al. 2004). Other studies of thyroid cancer and ^131^I exposure have been reviewed elsewhere (McNally et al. 2016; COMARE 2016). Factors that could be relevant to the risk of thyroid cancer following intakes of ^131^I include the level of stable iodine in the diets of those exposed, with iodine deficiency increasing the risk (Boice 2006; Zupunski et al. 2019; Shakhtarin et al. 2003).

### Interpretation of results

The question remains as to why this study did not detect an increased risk of thyroid cancer among persons exposed as young children (or *in utero*) to ^131^I from the 1957 Windscale fire in the most contaminated parts of Cumbria. This question is particularly pertinent in the light of the tentative prediction of Clarke (1989, 1990) that around eight cases of thyroid cancer would result from exposure to ^131^I released during the accident among those living in Cumbria within 50 km of Sellafield, a predicted number that would increase by ~ 30% using a later risk coefficient (ICRP 2007). Owing to the limited numbers of births during 1950–1958 in the contaminated areas and the consequent small expected numbers of cases, the resulting thyroid cancer incidence rates are based on small observed numbers of cases and have rather wide confidence intervals (Table 1), but the absence of evidence of excess cases is somewhat unanticipated given the number of cases suggested by Clarke (1989, 1990). It is possible that cases occurred among those exposed as older children or young adults, or others who could not be included in the study, or those lost to follow-up, for reasons discussed above. However, it seems unlikely that a sufficient number of cases has been missed for this to provide an adequate explanation, particularly since the majority of the predicted excess cases would occur among those exposed as young children.

In this respect, Clarke (1989, 1990) cautioned against placing undue reliance upon estimates of excess cases obtained from the application of nominal risk coefficients to collective doses composed of the sum of many small individual doses, a warning that has been emphasised elsewhere (ICRP 2007). Nominal risk coefficients have been derived making prudent assumptions about cancer risks following low-level exposures, assumptions made for the purposes of radiological protection (ICRP 2007) that could well overestimate such risks. Overprediction of expected thyroid cancer case numbers would artificially inflate the assessed statistical power of this study to detect an underlying raised risk. Of potential relevance is the absence of a discernible excess risk of thyroid cancer among those exposed in childhood to ^131^I discharged from the Hanford nuclear complex in Washington State during 1944–1957, a total discharge of ^131^I activity some 15 times greater than the release during the Windscale fire (Davis et al. 2004). Whatever the explanation, it can be inferred from the present study that it is unlikely the risk of thyroid cancer arising from exposure to ^131^I discharged during the Windscale accident of 1957 has been underestimated.

## Conclusions

This study has employed a large database of births in Cumbria from 1950 onwards to investigate the risk of thyroid cancer consequent to the 1957 Windscale accident. No increased incidence of thyroid cancer has been detected during 1981–2020 among those born in south-west Cumbria during 1950–1958, who are assessed to be at most risk of thyroid cancer from ^131^I released in October 1957 during the fire in one of the Windscale reactors at Sellafield. Observed numbers of cases in persons born in contaminated areas are small, but a substantially raised thyroid cancer risk in those most exposed to ^131^I can be excluded. It would be worthwhile continuing the follow-up of these birth cohorts beyond 2020, although this is very unlikely to alter the conclusions on thyroid cancer risk.

This absence of a discernible excess risk of thyroid cancer could be due to a number of factors. These include an overestimation of the thyroid doses received by children from intakes of ^131^I, but the comprehensive assessment of Crick and Linsley (1984) and the compatibility of predicted and measured thyroid doses, albeit based on limited data, suggests that any overestimation is not large. The swift action taken immediately after the accident to introduce a ban on the distribution of milk contaminated with ^131^I above a level that was calculated from information available in 1957 to deliver a dose to the thyroid of a child that exceeded 200 mGy undoubtedly reduced the collective thyroid dose received by Cumbrian children. The potential consequences of not taking such appropriate protective action are graphically illustrated by the reactor explosion at Chornobyl in 1986 when 1,800 PBq of ^131^I was released (UNSCEAR 2011) (1000 times greater than the discharge during the Windscale fire), when the authorities in the then USSR failed to prevent children in the most affected areas consuming milk heavily contaminated with radioiodine. This led to tens of thousands of children receiving thyroid doses > 1 Gy and thousands of additional thyroid cancer cases (UNSCEAR 2011, 2018). The present findings for Cumbria suggest that restricting the consumption of milk from farms near Sellafield was largely successful in its objectives.

Perhaps one of the more likely explanations for the absence of excess cases of thyroid cancer is that a calculation of case numbers based upon nominal risk coefficients derived for the purposes of radiological protection applied to collective doses obtained by aggregating many small individual doses does not produce realistic estimates of numbers of radiation-related cases of cancer. The assumptions underlying such a calculation do not have firm evidential foundations and estimates derived in this way are spurious (ICRP 2007).

The present study adds to the evidence base relating radiation exposure of the thyroid to the risk of thyroid cancer, specifically, the risk from low and moderate intakes of ^131^I by children. This is relevant to those areas contaminated with ^131^I released from the Fukushima Dai-ichi nuclear reactors in Japan during the accident in March 2011. Protective measures limited the thyroid doses assessed to have been received by the most exposed children (UNSCEAR 2022) and the findings of this and other studies encourage confidence in the validity of the conclusion of UNSCEAR (2022) that increased thyroid cancer risks will be small and unlikely to be detectable.

## Electronic supplementary material

Below is the link to the electronic supplementary material.

Supplementary Material 1

## Data Availability

The linkage exercise was conducted using the national cancer registries of England, Wales and Scotland and access to the confidential data held by the registries is restricted. Requests concerning the registration data held by the national cancer registries that were used in the linkage exercise conducted for this study should be directed to the registries. Queries concerning the Cumbrian births database should be directed to R.J.Q.McN.
