# Supplementary material for: Thyroid cancer incidence in cohorts exposed in childhood to 131I released during the Windscale nuclear reactor accident at Sellafield, England, in 1957
Source: Radiat Environ Biophys. 2024 Aug 16;63(4):491–503. doi: 10.1007/s00411-024-01087-y (PMC11588877; doi:10.1007/s00411-024-01087-y)
Supplement: Supplementary file 1 — Supplementary Material 1 [file 411_2024_1087_MOESM1_ESM.pdf]

Supplementary Information

for the paper published in *Radiation and Environmental Biophysics* in 2024 entitled

Thyroid cancer incidence in cohorts exposed in childhood to ^{131}I released during the Windscale nuclear reactor accident at Sellafield, England, in 1957

Richard J. Q. McNally[1*], Richard Wakeford[2*‡], Kathryn J. Bunch[3], Louise Hayes[1], Sally Vernon[4], Polly-Anne Jeffrey[4], Lizz Paley[4], Alex Elliott[5*]

[1] Population Health Sciences Institute & Newcastle University Centre for Cancer, Newcastle University, Sir James Spence Institute, Royal Victoria Infirmary, Queen Victoria Road, Newcastle upon Tyne, NE1 4LP, UK.

[2] Centre for Occupational and Environmental Health, Faculty of Biology, Medicine and Health, The University of Manchester, Oxford Road, Manchester, M13 9PL, UK.

[3] Formerly of the Childhood Cancer Research Group, Department of Paediatrics, University of Oxford, Headington, Oxford, OX3 9DU, UK.

[4] National Disease Registration Service, National Health Service England, 10 South Colonnade, Canary Wharf, London E14 4PU, UK

[5] School of Medicine, Dentistry and Nursing, University of Glasgow, Glasgow, G12 8QQ, UK

*These authors contributed equally to this work.

‡ Corresponding author: Richard.Wakeford@manchester.ac.uk

Birth Cohort	Number of Livebirths and Proportion of Female Births in Total Births			
	Area of ^{131}I contamination level in Cumbria			Total (all of Cumbria)
	Area 3 (lowest, reference)	Area 2 (intermediate)	Area 1 (highest)	
**1950-1958**	With linkable Cumbrian postcode and unique NHS numbers			
Male	26,169	2079	323	28,571
Female	25,208	1987	320	27,515
Total	51,377	4066	643	56,086
Female/Total (95% CI)	0.491 (0.486, 0.495)	0.489 (0.473, 0.504)	0.498 (0.459, 0.536)	0.491 (0.487, 0.495)
**1959-1980**	With linkable Cumbrian postcode and unique NHS numbers			
Male	64,656	5274	709	70,639
Female	61,247	4897	661	66,805
Total	125,903	10,171	1370	137,444
Female/Total (95% CI)	0.486 (0.484, 0.489)	0.481 (0.472, 0.491)	0.482 (0.456, 0.509)	0.486 (0.483, 0.489)

Supplementary Table S1. Numbers of male and female livebirths during 1950-1958 and 1959-1980 in three areas with different levels of ^{131}I contamination from the October 1957 Windscale accident, and the proportions of female births in total births in these six sub-cohorts. Numbers of livebirths are for those individuals in the Cumbrian births database with a known Cumbrian postcode, which were able to be linked to a unique NHS number used by the NHS in England and NHS Wales, providing an unambiguous linkage between a person in the Cumbrian births database and a person in the national databases (see details provided in Supplementary Table S2). The 95% confidence interval (CI) for the proportion of females in total livebirths is the Mid-P exact 95% CI obtained using the OpenEpi package (www.OpenEpi.com, accessed 20 November 2023) (see main text).

Birth cohort	Area*	Total database entries	Entries with NHS number linkage	Entries with unique NHS numbers
Total number of livebirths 1950-1980	All areas	206,703	194,440	194,273
Number of livebirths with no linkage to contamination area 1950-1980	Not known	794	748	743
1950-1958	Not known	329	300	296
1959-1980	Not known	465	448	447
Number of livebirths with linkable postcode 1950-1980	All areas	205,909	193,692	193,530
1950-1958	3	56,519	51,471	51,377
	2	4415	4074	4066
	1	689	644	643
	Total	61,623	56,189	56,086
1959-1980	3	132,269	125,956	125,903
	2	10,603	10,176	10,171
	1	1414	1371	1370
	Total	144,286	137,503	137,444

* The ^{131}I contamination area: Area 1, highest; Area 2, intermediate; Area 3, lowest (reference area)

Supplementary Table S2. Numbers of livebirths in 1950-1980, and in 1950-1958 and 1959-1980, recorded in the Cumbrian births database with a Cumbrian postcode that links to one of the three areas with different levels of ^{131}I contamination from the October 1957 Windscale accident. Livebirth numbers are tabulated using three counts: unique entries in the Cumbrian births database, unique entries in the Cumbrian births database that can be linked to an NHS number used by the NHS in England and NHS Wales, and entries that can be linked to a unique NHS number (since multiple entries are occasionally linked to the same NHS number).
